# Response to a letter on “Physiological effects of high-intensity versus low-intensity noninvasive positive pressure ventilation in patients with acute exacerbation of chronic obstructive pulmonary disease: a randomised controlled trial”

**DOI:** 10.1186/s13613-022-01041-5

**Published:** 2022-07-11

**Authors:** Zujin Luo, Zhixin Cao, Chen Wang

**Affiliations:** 1 Xuanwu Hospital, Capital Medical University, No. 45 Changchun Street, Xicheng District, Beijing, 100053 China; 2 Department of Respiratory and Critical Care Medicine, Beijing Institute of Respiratory Medicine, Beijing Chao-Yang Hospital, Capital Medical University, No. 5 Jingyuan Road, Shijingshan District, Beijing, 100043 China; 3 Department of Pulmonary and Critical Care Medicine, China-Japan Friendship Hospital, No. 2 Yinghua East Street, Chaoyang District, Beijing, 100029 China; 4 National Clinical Research Center for Respiratory Diseases, Beijing, China; 5 Chinese Academy of Medical Sciences and Peking Union Medical College, Beijing, China; 6 Department of Respiratory Medicine, Capital Medical University, Beijing, China

Dear editor

We appreciate Toumi et al. for their interest, comments, and suggestions on our article, titled “Physiological effects of high-intensity versus low-intensity noninvasive positive pressure ventilation in patients with acute exacerbation of chronic obstructive pulmonary disease: a randomised controlled trial” [1], and thank you for the opportunity to respond to the letter. Our responses are as follows.

We always pay attention to change in arterial pH, and in this trial recorded it 2, 6, 24, 48, and 72 h after randomisation. Because of the sharp decrease in elevated arterial carbon dioxide tension (PaCO_2_), there would be secondary metabolic alkalosis due to renal compensation. In our experience, in most cases, this kind of transient alkalosis does not have adverse effects. However, a previous study reported that a pH > 7.55 can have a detrimental effect on patient outcomes [2]. Hence, we defined severe alkalosis as pH > 7.55, and arginine was provided if that threshold was met in our trial protocol. Although pH 24 h after randomisation differed significantly between the two groups, the mean pH was only 7.48 in the high-intensity noninvasive positive pressure ventilation (NPPV) group. Moreover, no severe alkalosis occurred, no arginine was required, and no adverse effects (including seizures) were observed in our trial. Due to high-pressure support with prolonged NPPV duration, we did not observe compensatory hypoventilation and the consequent increase in PaCO_2_ in the high-intensity NPPV group.

We agree that it is of great importance to obtain a pH > 7.35 for NPPV success. However, previous evidence indicates that low-intensity NPPV would fail in approximately 15% of patients with acute exacerbation of chronic obstructive pulmonary disease (AECOPD) [3]. NPPV failure in such patients may be partly associated with inadequate pressure support provided by low-intensity NPPV through several possible mechanisms. First, despite the decrease in PaCO_2_ in the majority of patients, this decrease is limited, and PaCO_2_ can be difficult to normalise. PaCO_2_ can easily increase if the patient’s clinical conditions worsen, which could trigger NPPV failure [4]. Second, in a small number of patients with the most severe respiratory mechanics, PaCO_2_ can continuously worsen until NPPV failure. Third, in some individuals NPPV is poorly tolerated and must be discontinued, possibly because of inadequate pressure support, which ultimately leads to NPPV failure and subsequent endotracheal intubation. Fourth, unimproved hypercapnia is unbeneficial for improving sodium and fluid retention (and thus unbeneficial for improving airway oedema and respiratory mechanics) and might compromise the strength and endurance of the diaphragm, which in turn would prevent a decrease in PaCO_2_ [5]. Our aim was to explore the physiological effects of high-intensity NPPV compared to low-intensity NPPV in patients with AECOPD.

In our trial, a V60 noninvasive ventilator (Philips Respironics, Carlsbad, CA, USA) was used for all patients. According to our experience, a rise slope at level 1 or 2 is proper for AECOPD patients. In our trial protocol, the rise slope was set at level 1 or 2 in both groups. An important characteristic of the V60 Ventilator, called Auto-Trak sensitivity, is its ability to automatically adjust its triggering and cycling algorithms to maintain optimum performance in the presence of leakage. Hence, the ventilator does not require us to set triggering and cycling sensitivity, but they were equally used in the two groups.

Intrinsic positive end-expiratory pressure could be accurately measured using an end-expiratory occlusion manoeuvre in invasively ventilated patients. However, because of circuit leakage and possible glottis closure, it is difficult to measure it in patients receiving NPPV. Also because of the lack of reliable monitoring of expiratory flow and the unavoidable leakage during NPPV, we cannot guarantee an accurate estimation of airway obstruction by analysing expiratory flow curves.

Our trial was carried out with this specialised noninvasive ventilator, which could compensate for leakage. Theoretically, high-intensity NPPV could be performed by intensive care unit ventilators with a double-limb circuit if they feature leakage compensation. However, further study is required to address this issue.

## Data Availability

None.

## Abbreviations

AECOPD: Acute exacerbation of chronic obstructive pulmonary disease
NPPV: Noninvasive positive pressure ventilation
PaCO_2_: Arterial carbon dioxide tension.
